# The sensory gene repertoire of deep-sea hydrothermal shrimp

**DOI:** 10.1371/journal.pone.0354016

**Published:** 2026-07-15

**Authors:** Thomas Chertemps, Nicolas Montagné, Adrien Mathou, Nelly Léger, Magali Zbinden, Juliette Ravaux

**Affiliations:** 1 Institut d’Écologie et des Sciences de l’Environnement de Paris (iEES-Paris), Sorbonne Université, Université Paris Cité, Université Paris Est Créteil, CNRS, IRD, INRAE, Paris, France; 2 Institut Universitaire de France (IUF), France; 3 Laboratoire de Biologie des Organismes et des Écosystèmes Aquatiques (BOREA), Sorbonne Université, MNHN, CNRS, IRD, UA, Paris, France; Sao Paulo State University Julio de Mesquita Filho: Universidade Estadual Paulista Julio de Mesquita Filho, BRAZIL

## Abstract

Deep-sea hydrothermal vents are among the most extreme and geochemically dynamic environments on Earth, characterized by steep gradients in temperature and chemical concentration. Alvinocaridid shrimp dominate the macrofaunal biomass at these sites and rely on their sensory systems to navigate these dark habitats. In this study, we provide the first comprehensive characterization of sensory gene repertoires associated with chemical and thermal detection in four deep-sea shrimp species occupying different ecological niches at hydrothermal vents on the Mid-Atlantic Ridge: *Rimicaris exoculata*, *Rimicaris chacei*, *Mirocaris fortunata*, and *Alvinocaris markensis*. Through *de novo* transcriptome assembly of sensory organs, we identified a great expansion of the variant Ionotropic Receptor (IR) family. Notably, we annotated 442 candidate IR transcripts in *R. exoculata*, the largest repertoire documented in any decapod crustacean. This expansion is primarily driven by the IR40a clade, suggesting adaptive radiation centred on the detection of the complex chemical waterscape of vents. Furthermore, we have identified a diverse set of Transient Receptor Potential (TRP) channels, including 18 TRPA genes in *R. exoculata*, which likely facilitate fine-tuned thermosensation in a high-gradient environment. In contrast, Chemosensory Proteins and Niemann-Pick type C2 proteins exhibited ubiquitous expression patterns, suggesting broader physiological roles beyond olfaction. These findings offer insights into the molecular adaptations that enable Alvinocarididae to thrive in the deep sea.

## Introduction

The deep sea, covering more than 70% of the Earth’s surface, is the largest and one of the least explored ecosystems on the planet. Among its most remarkable habitats are hydrothermal vent ecosystems, which were discovered in 1977 along the Galápagos Rift [1]. These environments rank among the most extreme and geochemically dynamic on Earth. They are characterized by steep temperature gradients (from 2°C ambient seawater to vent fluids exceeding 400°C), high hydrostatic pressure, heavy metal toxicity and high levels of reduced chemical compounds, such as hydrogen sulfide (H_2_S), methane and hydrogen. Despite the seemingly inhospitable conditions, hydrothermal vents host a wide range of unique animals that have developed remarkable physiological and biochemical adaptations that enable their survival [2]. Most notably, these ecosystems rely on chemolithoautotrophic bacteria that oxidize reduced chemical compounds and form the basis of the food web [3–5]. The vent fauna is dominated by species that form symbiotic relationships with chemosynthetic bacteria [6–8].

Among the diverse fauna endemic to hydrothermal vents, alvinocaridid shrimp (family Alvinocarididae) dominate the macrofaunal biomass at many vent sites. These shrimps face the critical challenge of locating food, interacting with their conspecifics, and selecting microhabitats suited for their survival in an environment devoid of sunlight [9]. The sensory modalities underpinning these behaviours are likely multiple but remain poorly understood. Vision has been mainly studied in *Rimicaris exoculata*, which lives in dense swarms on chimney walls close to the hydrothermal emissions. Its modified, highly sensitive eyes may allow detection of the faint light emitted by hot fluids [10]. Chemoreception has been investigated in *R. exoculata* and *Mirocaris fortunata*, and the current data are summarized in [11]. The anatomy of the olfactory system does not present any particular features that suggest an enhanced sense of smell in these species [11–13]. While *M. fortunata* detects sulfide, a typical compound of hydrothermal fluids, using its antennal appendages, neither *M. fortunata* nor *R. exoculata* exhibits attraction to sulfide sources [11,14]. However, both species show a pronounced attraction to warm water sources, highlighting thermosensation as a key modality for locating vents [11,15,16]. To further investigate the sensory modalities at play in these extreme environments, the present study explored the repertoire of genes potentially involved in chemical and thermal sensing in shrimp endemic to hydrothermal vents from the Mid-Atlantic Ridge.

Chemoreception in decapod crustaceans occurs in sensilla mainly located on the antennal appendages, with the aesthetascs dedicated to olfaction on the lateral flagellum of the antennule (the first pair of antennules), and the other sensilla involved in chemo- and mechanodetection on both pairs of antennae [17–20]. Stimulus detection is achieved by sensory neurons, whose membranes contain receptor proteins responsible for signal transduction. The molecular basis of chemoreception in crustaceans has essentially been studied in large decapods, such as lobsters, and apart from a few model species, the repertoires of candidate sensory receptors are still largely unknown [21–23]. In crustaceans, three major multigenic families encode candidate sensory receptors: variant Ionotropic Receptors (IRs), Gustatory Receptors (GRs) and Transient Receptor Potential channels (TRPs). All of these have been discovered and mainly studied in insects but are expected to exhibit similar functions in other pancrustaceans [21,24,25]. Beyond the detection of chemicals, it has been demonstrated that IRs and TRPs also function as environmental sensors, detecting temperature and humidity in the antennae of the fruit fly *Drosophila* [26,27]. It is likely that receptors located in the antennules of crustaceans also perform thermal detection [19].

IRs have evolved from ionotropic glutamate receptors (iGluRs) in the last common ancestor of protostomes [28]. They mainly function as chemoreceptors in both taste and olfaction, but also as temperature and moisture sensors [29]. IRs contain three transmembrane domains and an extracellular ligand-binding domain. They associate into heterotetramers to act as stimulus-gated ion channels, including highly conserved co-receptors (IR25a, IR8a, IR76b and IR93a) that are necessary for receptor function and more divergent tuning IRs that provide specificity to the receptor complex [30–32]. Current knowledge suggests that IRs are the major chemoreceptors in decapods [23,33,34]. GRs belong to the large superfamily of 7-transmembrane domain ion channels and are found in all arthropods [35,36]. They assemble into homo- or heterotetramers that form ligand-gated ion channels [37]. They are the main taste receptors in insects, but their function outside insects remains largely unknown. Their limited number in decapods suggests a limited, yet possible, role in chemoreception [34]. Lastly, TRP channels are an ancient and extremely diverse superfamily composed of nine families in animals [38]. They are proteins containing six transmembrane domains assembled into tetramers to form multimodal cation channels that can be activated by physical and chemical stimuli [39,40]. They act as molecular sensors for a wide range of environmental factors, including temperature, pH, osmolarity, mechanical forces and chemical compounds [25,26,41]. In extreme environments such as hydrothermal vents, TRP channels may play a crucial role in sensing thermal and chemical gradients.

Chemical senses involve not only chemoreceptors responsible for signal transduction but also binding proteins capable of transporting chemicals to the receptors. In arthropods, the so-called odorant-binding proteins are restricted to insects, and other families of soluble proteins have been proposed to participate in chemoreception, particularly chemosensory proteins (CSPs) and Niemann-Pick type C2 proteins (NPC2) [42]. CSPs are small proteins characterized by the presence of four invariant cysteine residues. In insects, they can bind to different hydrophobic compounds, among other functions, thereby making chemoreceptors more sensitive and specific [43–45]. NPC2 proteins facilitate the transport and metabolism of lipids and other hydrophobic molecules and may play a significant role in the trafficking of chemosensory signals [46,47].

**Fig 1:**
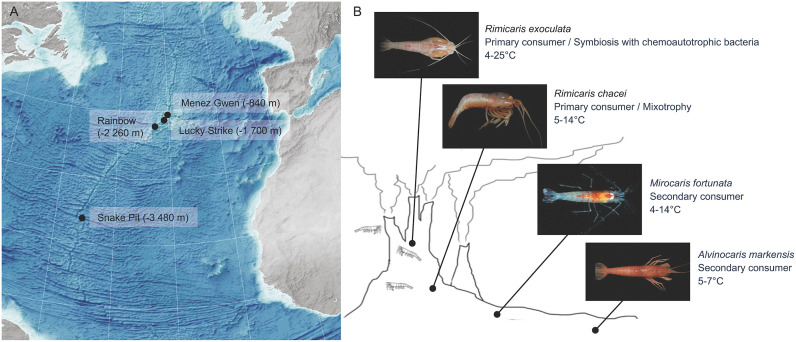
Sampling sites and alvinocaridid species. (A) Map showing the location of hydrothermal vent sites on the Mid-Atlantic Ridge where shrimp were collected for this study (vent site depth is given in brackets). Map modified from NASA Earth Observatory (https://assets.science.nasa.gov/content/dam/science/esd/eo/images/imagerecords/87000/87189/midatlantic_mdl_2014_bathy_lrg.png) (B) Distribution of the four shrimp species in the hydrothermal ecosystem and in the trophic web, mean temperature in shrimp microhabitat. References for mean temperature data: *R. exoculata* [58], *M. fortunata*, *R. chacei* and *A. markensis* [53]. See the text for other references. Credits: *Rimicaris exoculata*: BICOSE 2024/A. Mathou; *Rimicaris chacei*: [href:http://coldb.mnhn.fr/catalognumber/mnhn/iu/2013-15618]http://coldb.mnhn.fr/catalognumber/mnhn/iu/2013-15618; *Alvinocaris markensis:* [href:http://coldb.mnhn.fr/catalognumber/mnhn/iu/2013-15609]http://coldb.mnhn.fr/catalognumber/mnhn/iu/2013-15609; *Mirocaris fortunata*: Océanopolis/D. Barthélémy.

Over the last ten years, RNA sequencing (RNA-seq) and de novo transcriptome assembly have transformed our ability to investigate the molecular basis of adaptation in non-model organisms, especially those from extreme environments [48–50]. Here, we generated transcriptome data from the sensory organs of four deep-sea vent shrimp species, all belonging to the Alvinocarididae family but occupying distinct niches in the hydrothermal ecosystems of the Mid-Atlantic Ridge (Fig 1). *Rimicaris exoculata*, the most abundant species, lives in dense swarms on the chimney walls close to the hot fluid emissions to feed its symbiotic chemoautotrophic bacterial community [9]. The less abundant *Rimicaris chacei* also lives on the chimneys and harbours a bacterial symbiotic community, although less developed than in *R. exoculata* [51]. It can also be found on sulfide blocks in areas of weak fluid emissions [52,53]. *R. chacei* has a mixed diet, consisting partly of carbon from its associated symbiotic bacteria and partly from other food sources [54]. The third species, *Mirocaris fortunata*, colonizes habitats slightly further from the chimneys, among mussel beds in colder, diffuse currents. It is reported to be a predator or scavenger [16,55,56]. The fourth species, *Alvinocaris markensis*, is found as solitary individuals typically located at the base of active edifices and in mussel assemblages, and is also considered a predator or scavenger [52,57]. Our objectives were to explore the diversity of chemosensory gene repertoires in these shrimps by employing *de novo* transcriptome assembly, phylogenetic analysis and tissue-specific expression profiling. We aimed to identify species-specific variations that may indicate ecological niche differentiation and evaluate expression patterns across chemosensory organs to deduce functional specialization.

## Materials and methods

### Animal collection

Adult specimens of four shrimp species were collected at hydrothermal sites along the Mid-Atlantic Ridge: *Rimicaris exoculata* Williams & Rona, 1986, *Rimicaris chacei* Williams & Rona, 1986, *Alvinocaris markensis* Williams, 1988, *Mirocaris fortunata* Martin & Christiansen, 1995 (see Fig 1A and Table 1 for cruises and sites). Specimens of alvinocaridid shrimp were collected with the suction device of either the Remotely Operated Vehicle ‘Victor 6000’ or the Diving Support Vessel ‘Nautile 6000’, operating from the Research Vessel ‘Pourquoi Pas?’ or ‘L’Atalante’. Immediately after collection, three specimens of each species were dissected, and the organs of interest were instantly frozen in liquid nitrogen.

**Table 1:** List of samples.

Species	Sample	Cruise	Site	Depth (m)	Location
*Rimicaris exoculata*	A1 MF	BIOBAZ 2013	Rainbow	2260	36°13'N, 33°54'W
	A1 LF				
	A2				
	Abdominal muscles				
	Mxp2,3 and P1	BICOSE 2014	Snake Pit	3480	23°23'N, 44°58'W
	P5				
*Rimicaris chacei*	A1 MF and LF	BIOBAZ 2013	Rainbow	2260	36°13'N, 33°54'W
	A2				
	Abdominal muscles				
*Alvinocaris markensis*	A1 MF and LF	BIOBAZ 2013	Rainbow	2260	36°13'N, 33°54'W
	A2				
	Abdominal muscles				
*Mirocaris fortunata*	A1 MF and LF	MOMARSAT 2016	Lucky Strike	1700	37°17'N, 32°16'W
	A2				
	Abdominal muscles	BIOBAZ 2013	Menez Gwen	840	37°51'N, 31°31'W

A1 MF—medial flagellum of antennule, A1 LF—lateral flagellum of antennule, A2—second antenna, Mxp2,3—second and third pairs of maxillipeds, P1—first pair of walking legs, P5—fifth pair of walking legs. References for the oceanographic missions are as follows: doi.org/10.17600/13030030 (BIOBAZ 2013), doi.org/10.17600/16001200 (MOMARSAT 2016) and doi.org/10.17600/14000100 (BICOSE 2014). Authorization to access and collect samples from sites located in the Portuguese EEZ was issued by the Ministério dos Negócios Estrangeiros and the Região autónoma dos Açores.

### Sample preparation and RNA extraction

**Fig 2:**
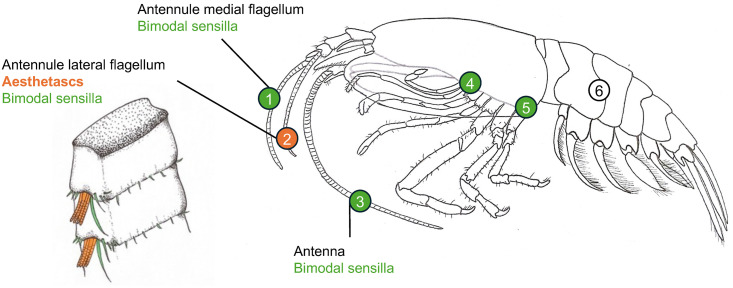
*Rimicaris exoculata* tissue sampling for transcriptome analysis. 1—medial flagellum of antennule (A1 MF), 2—lateral flagellum of antennule bearing aesthetascs (A1 LF), 3—second antenna (A2), 4—second and third pairs of maxillipeds (Mxp2,3) and first pair of walking legs (P1), 5—fifth pair of walking legs (P5), 6 —abdominal muscles. Orange dot: samples containing aesthetascs involved in olfaction; Green dot: samples containing bimodal chemo- and mechanosensory sensilla mediating distributed chemoreception; White dot: samples containing no sensilla.

The tissues collected from each species are detailed in Fig 2 and Table 1. For *R. exoculata*, *R. chacei* and *A. markensis*, three individuals were dissected during the BIOBAZ 2013 campaign to collect the A1 pair (separating the median and lateral flagella for *R. exoculata*), the A2 pair, and abdominal muscle tissue. For *R. exoculata*, additional samples were collected from three other individuals collected during the BICOSE 2014 campaign: the Mxp2, Mxp3, P1 and P5 pairs. For *M. fortunata*, the A1 and A2 pairs were collected from three individuals during the MOMARSAT 2016 campaign, and the abdominal muscle samples came from three other individuals collected during the BIOBAZ 2013 campaign. Each pool of tissues (from three different individuals) was used to generate one library per species and per tissue, i.e., a total of 15 libraries.

For each library, RNA was extracted by homogenizing frozen tissues in tubes containing TRIzol^TM^ Reagent (Thermo Fisher Scientific, Waltham, MA, USA) and a mixture of micro-ceramic beads using a MiniLys® homogenizer (Bertin Technologies SAS, Montigny Le Bretonneux, France). The liquid phase was separated from the beads, and total RNA was extracted using the phenol/chloroform method, as described in the TRIzol^TM^ Reagent user guide. Total RNA was treated with TURBO DNase (Thermo Fisher Scientific) according to the manufacturer’s instructions, then purified and concentrated using the RNeasy MinElute^TM^ Cleanup Kit (Qiagen, Hilden,Germany). RNA quality and quantity were checked by spectrophotometry and using an Experion^TM^ Automated Electrophoresis Station (BioRad). Total RNA extracted from each tissue type of each species was frozen in liquid nitrogen and stored in aliquots at −80°C.

### RNA sequencing, de novo transcriptome assembly and transcript abundance

cDNA library construction and sequencing were performed using the high-throughput sequencing platform MGX-Montpellier GenomiX (Montpellier, France). Libraries were prepared using the Illumina TruSeq Stranded mRNA kit. Sequencing (250-bp paired-end reads) was performed on an Illumina HiSeq 2500 using the TruSeq Rapid SBS kit. The initial assessment of the quality of the raw reads was conducted using the FastQC v0.72 tool [59], and low-quality reads were trimmed using the Trimmomatic v0.36.6 tool [60]. The following parameters were used: sliding window = 4; average quality = 20; minlen = 30 bases; headcrop = 10 bases; and trailing minimum quality = 20. A de novo reference transcriptome assembly was then conducted for each species, mixing reads from all samples using Trinity v2.8.4 [48]. We set a minimum contig length of 200 and a minimum count of 1 for the K-mers to be assembled.

Protein-coding sequences were extracted from the reference transcriptome using Transdecoder v5.5.0 [49], setting a minimum protein length of 50 amino acids. Redundant sequences were clustered using CD-HIT EST v1.2 [61] with a similarity threshold of 0.9 and a word size of 8 (data metrics summarized in S1 Table). The completeness of the assembled transcriptomes was assessed using BUSCO v4.1.4 [62], which tests the assembly for the presence of 1,013 single-copy orthologs that are highly conserved in arthropods (arthropoda_odb10). Functional annotation of the coding sequences was performed by searching the protein domain databases Pfam, PANTHER and SUPERFAMILY using InterProScan 5 [63] and by searching the best hit in the NCBI ‘nr’ amino acid sequence database using DIAMOND [64]. All sequences have been deposited in the ENA database under accession number PRJEB113328.

To measure expression levels, clean reads were mapped on each related reference transcriptome using Kallisto v0.46.2 via pseudoalignment of corrected reads [65]. The total number of filtered reads was normalized using the trimmed mean of M-values (TMM) method [66]. Transcript abundance was measured in each sample as transcripts per million (TPM). The log_2_(TPM + 1) values were used to generate expression heatmaps using the TBtools suite [67].

### Identification of sensory genes

For the five sensory gene families to be analysed, datasets containing amino acid sequences annotated in six other pancrustacean species were first created: *Drosophila melanogaster*, *Daphnia pulex*, *Panulirus argus*, *Cherax quadricarinatus*, *Penaeus chinensis* and *Macrobrachium nipponense*. Sequences annotated in the reference genomes of the vinegar fly *D. melanogaster* and the water flea *D. pulex* were found in [24] (IRs, GRs, CSPs and NPC2s) and [22] (TRPs). Sequences identified in the transcriptome of the spiny lobster *P. argus* were from [34] (IRs) and [22] (TRPs). In addition, we retrieved sequences automatically annotated in the reference genomes of the crayfish *C. quadricarinatus* (assembly GCA_038502225.1), the prawn *P. chinensis* (GCA_019202785.2) and the palaemonid shrimp *M. nipponense* (GCA_015104395.2) from GenBank. For IRs and TRPs, we also included sequences from the shrimp *Palaemon carinicauda* (GCA_036898095.2) and the lobster *Homarus americanus* (GCA_018991925.1; [23]). These amino acid sequences were used as queries to search the reference transcriptomes of the four hydrothermal shrimp species using NCBI tBLASTn as implemented into Galaxy [68] with an e-value cutoff set at 1e-20. In parallel, the results of the InterProScan analysis were mined for the following Pfam protein domains: PF00060, Ligand-gated ion channel (IRs); PF08395, 7tm_7 chemosensory receptor (GRs); PF03392, OS-D Insect pheromone-binding family (CSPs); PF06011, Transient receptor potential ion channel; and PF08344, Transient receptor ion channel II (TRPs). To verify the annotations, sequences were searched against the NCBI nr database using BLASTp. Redundant unigenes encoding the same protein but not clustered by CD-HIT-EST were clustered consolidated.

### Sequence alignment and phylogenetic analysis

Amino acid sequences were aligned using MAFFT (with the L-INS-i option) [69]. Phylogenetic trees were constructed using PhyML with the nearest neighbour interchange (NNI) method [70], based on the best substitution model determined by the SMS server [71]. Branch support was estimated using a Bayesian-like transformation of aLRT (aBayes) [72]. Dendrograms were created and annotated using Figtree v1.4.2.

## Results

### Assembly of reference transcriptomes

The RNA-seq approach generated substantial datasets ranging from 7.3 million cleaned reads (*A. markensis* abdominal muscle) to 80.3 million cleaned reads (*R. exoculata* P5 legs), with a cleaning efficiency exceeding 95% in all samples (Supplementary Table S1). De novo assembly produced transcriptomes of varying complexity, with contig numbers spanning from 224,046 (*R. chacei*) to 621,742 (*M. fortunata*), and unigene counts from 71,225–128,893 (number of CDs yielding a protein at least 50 a.a. in length). BUSCO assessments revealed high transcriptome completeness, with *R. exoculata* achieving 89.7% complete ortholog representation, followed by *M. fortunata* (82.2%), *A. markensis* (77.1%) and *R. chacei* (72.5%). These metrics demonstrate a robust assembly quality suitable for comprehensive gene family analysis.

### Diversity and expression pattern of candidate chemoreceptors

**Fig 3:**
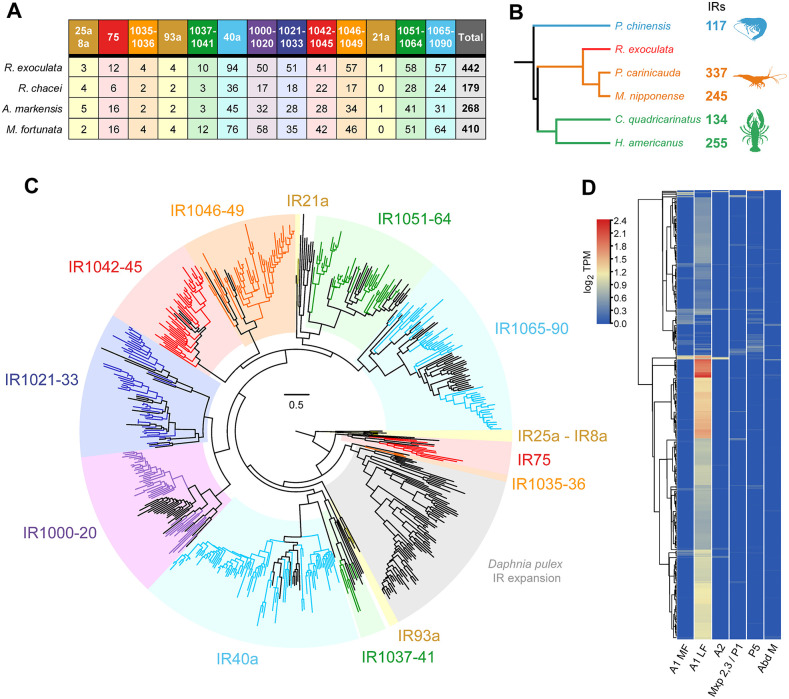
Phylogenetic tree and tissue expression of IRs in four hydrothermal shrimp. (A) Number of transcripts identified for each of the 13 phylogenetic lineages of variant Ionotropic Receptors (IRs) in the reference transcriptomes of the hydrothermal shrimp species. (B) Number of IR genes found in reference genomes of decapods from the suborders Dendrobranchiata (blue), Caridea (orange) and Astacidea (green). The tree topology and branch lengths were derived from [73]. (C) Maximum-likelihood phylogeny of pancrustacean IRs based on amino acid sequences identified in the transcriptome of *Rimicaris exoculata*, the transcriptome of the spiny lobster *Panulirus argus* [34], and the genome of the water flea *Daphnia pulex* (Dpul, [24]). Branches corresponding to *R. exoculata* IRs are shown in color. IR lineages were named according to [22]. The scale bar indicates the expected number of amino acid substitutions per site. (D) Heatmap showing the expression levels of IR transcripts in *R. exoculata*, measured as transcripts per million (TPM) in six tissues (medial flagellum of the antennules, A1 MF; lateral flagellum of the antennules, A1 LF; second antennae, A2; mix of second and third maxillipeds plus first walking legs, Mxp2,3/P1; fifth walking legs, P5; abdominal muscle, Abd M). The raw TPM values are listed in S2 Table.

The most promising candidates for chemosensory receptors in decapods are variant ionotropic receptors (IRs). Given the higher number of sequenced libraries in *R. exoculata* (six different tissues compared to three in the other species), we initiated our search for the gene sequences of interest in this species. This search, conducted by sequence identity with IRs from other arthropods and by protein motif, allowed us to identify an extraordinary diversity of IRs in the *R. exoculata* reference transcriptome, with 442 sequences (Fig 3A). This included sequences corresponding to the conserved IR coreceptors IR25a, IR8a and IR93a, but not IR76b. Due to the small size of these sequences, it is often impossible to determine which might correspond to a single gene, making it challenging to provide an exact estimate of the number of IR genes. Nevertheless, this is likely the largest number of IRs identified in a decapod crustacean. In comparison, the *P. argus* transcriptome contains 252 sequences encoding variant IRs [22]. To further this comparison, we also mined the available reference genomes of decapods and found that IR diversity in the vent-endemic species surpassed that documented in non-vent decapods (Fig 3B), including 245 IR genes in the freshwater shrimp *Macrobrachium nipponense*, 337 in the estuarine shrimp *Palaemon carinicauda* (suborder Caridea, similar to hydrothermal shrimp), 117 in the prawn *Penaeus chinensis* (Dendrobranchiata), 255 in *Homarus americanus* and 134 in the crayfish *Cherax quadricarinatus* (suborder Pleocyemata). Phylogenetic analysis conducted with these sequences, along with *R. exoculata* IRs (Fig 3C), revealed that the increase in number resulted from gene expansions in 7 out of the 13 IR clades previously described [22]. Such expansions were particularly pronounced in the IR40a clade, which contained 94 *R. exoculata* sequences. The expression of IRs in *R. exoculata* was primarily restricted to the lateral flagellum of the antennules (Fig 3D), the main chemosensory organ of decapods [19]. In the other three species, we identified 179 IR transcripts in *R. chacei*, 268 in *A. markensis*, and 410 in *M. fortunata* (Fig 3A). As with *R. exoculata*, we did not identify any IR76b sequences in these species, nor any IR8a sequences in *R. chacei*. The expression of most, if not all, IRs was restricted to the antennules, although the medial and lateral flagella were not separated in these cases (S1 Fig).

In addition to IRs, another family of candidate chemoreceptors in arthropods is the Gustatory Receptor (GR) family. Here, we found only two transcripts encoding GRs per species in *R. exoculata*, *R. chacei,* and *A. markensis* and none in *M. fortunata*. They belong to three different gene lineages, GR1, 2 and 3, which are also present in *M. nipponense* and *P. chinensis* (S2A Fig). In the three hydrothermal species, GR2 was the most highly expressed gene in both antennules and antennae (S2B Fig).

### Candidate binding proteins involved in chemoreception

By analysing the four reference transcriptomes, we identified CSP transcripts belonging to seven different lineages, some of which likely correspond to species-specific duplicates (S3A Fig). The number of CSPs identified for each species ranged from four in *M. fortunata* to eight in *R. exoculata*. Interestingly, some CSPs were clearly overexpressed in appendages compared to muscle tissues, namely CSP7 and CSP2 (*R. exoculata*, antennal appendages, maxillipeds and locomotory appendages), CSP5 and CSP3 (*R. chacei,* antennal appendages), CSP5.2 (*A. markensis*, antennules) and CSP4 (*M. fortunata*, antennal appendages) (S3B Fig). We also identified members of five distinct NPC2 lineages, from two in *R. chacei* to seven in *R. exoculata* (S4A Fig). Four of these lineages were not specific to hydrothermal shrimp, as they contained sequences annotated from the genomes of *M. nipponense*, *P. chinensis,* or *C. quadricarinatus*. However, members of the NPC2_3 lineages were identified only in *R. exoculata* (three sequences), with no annotated homologue in other decapods. The expression of NPC2 was not restricted to antennal appendages and did not show any particular pattern related to chemical function (S4B Fig).

### Genes potentially involved in multiple sensory modalities

**Fig 4:**
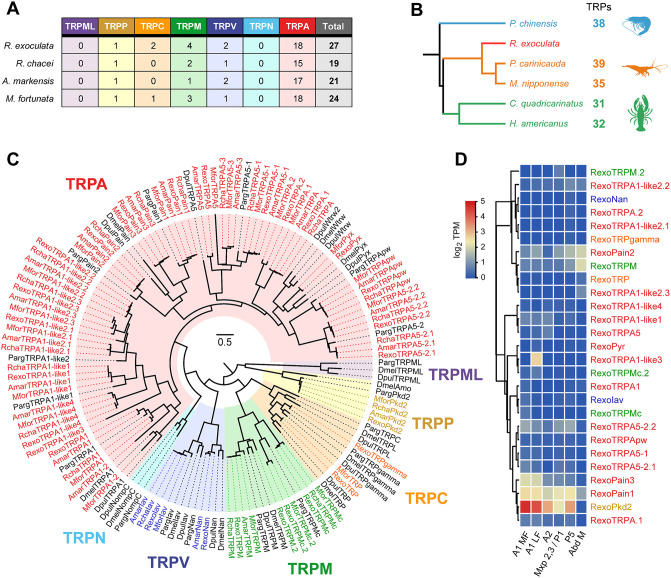
Phylogenetic tree and tissue expression of TRPs in four hydrothermal shrimp. (A) Number of transcripts identified for each of the seven families of Transient Receptor Potential channels (TRP) in the reference transcriptomes of the hydrothermal shrimp species. (B) Maximum-likelihood phylogeny of pancrustacean TRPs, based on amino acid sequences identified in the transcriptomes of the hydrothermal shrimp species (*Alvinocaris markensis*, Amar; *Mirocaris fortunata*, Mfor; *Rimicaris chacei*, Rcha; *Rimicaris exoculata*, Rexo), in the transcriptome of the spiny lobster *P. argus* (Parg) and in the genomes of the water flea *D. pulex* (Dpul) and the fly *Drosophila melanogaster* (Dmel). Sequences were retrieved from Kozma et al. (2020). The colors show the different TRP classes. The scale bar indicates the expected number of amino acid substitutions per site. (C) Number of TRP genes found in reference genomes of decapods from the suborders Dendrobranchiata (blue), Caridea (orange) and Astacidea (green). (D) Heatmap showing the expression levels of TRP transcripts in *R. exoculata*, measured as transcripts per million (TPM) in six tissues (as described in Fig 3). Transcript names are color-coded according to the TRP class, as in (B). The raw TPM values are listed in S2 Table.

TRPs are membrane channels that are involved in numerous sensory functions. As with the other families, we searched for sequences first in *R. exoculata* and then in the three other hydrothermal species. We identified 42 transcripts in *R. exoculata*, corresponding to 27 different genes (Fig 4A). This value is comparable to or slightly lower than those reported for other decapod reference genomes, such as *Macrobrachium nipponense* (35), *Homarus americanus* (32), *Penaeus chinensis* (38), *Cherax quadricarinatus* (31) and *Palaemon carinicauda* (39) (Fig 4B). Phylogenetic analysis revealed that these 27 *R. exoculata* TRPs belong to five of the seven TRPs families found in Metazoa (Fig 4A-C). No TRPML or TRPN was identified. TRPAs, several of which are implicated in thermoreception in animals, exhibited the highest diversity, with 18 genes identified. This is twice the number found in the spiny lobster *Panulirus argus* [22]. The phylogeny showed instances of possible gene duplications, for example, concerning the genes encoding TRPA1-like.2 and Pain (Fig 4C). Quantification of gene expression levels showed an overall low expression of TRPs in the different tissues, with the exception of Pkd2, which was expressed at very high levels in all appendages, including the first and second pairs of antennae, maxillipeds and legs (Fig 4D). Pain1 and Pain3 were also found to be expressed at relatively high levels compared to other TRPs, with expression restricted to the antennules (Pain3) or the appendages (Pain1). The subsequent search for TRP sequences in the other three hydrothermal shrimp species yielded similar results, with 19–24 genes and the same diversity within the TRPA family (Fig 4A-C). Similar to *R. exoculata*, Pkd2 and Pain1 were highly expressed in the two pairs of antennae of these species (S5 Fig). In contrast to the extensive IR gene expansions observed in hydrothermal species, TRP gene diversity appears to be relatively conserved across decapod lineages, indicating distinct evolutionary trajectories for these two chemosensory receptor families.

## Discussion

**Fig 5:**
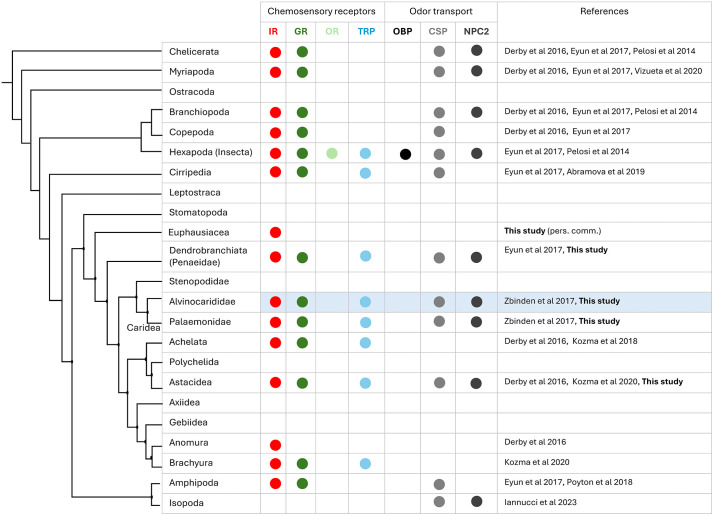
Proteins involved in chemosensory detection and chemical transport, known to be expressed in each clade of arthropods. Updated data from [21], derived from *de novo* transcriptome assemblies and reference genomes. Phylogeny adapted from [79].

The deep-sea hydrothermal environment has driven evolutionary changes that have moulded the sensory systems of endemic fauna. Active vents emit superheated fluids (up to 350°C) that mix with the cold abyssal surrounding seawater (around 4°C) to produce turbulent zones with steep chemical and thermal gradients over short distances and periods of time [56,74–77]. These conditions likely affect chemosensory perception, requiring enhanced sensitivity to navigate complex chemical and thermal signatures while maintaining functionality under extreme physicochemical stress. Previous studies have shown that hydrothermal shrimp have functional sensory systems for detecting chemical compounds, such as food or hydrogen sulfide contained in hydrothermal fluids [14], dim light produced by infrared radiation from hot fluids [78], and temperature (behavioral attraction to hot water sources; [11,15]. Here, we investigated the molecular repertoire associated with chemo- and thermodetection in shrimp species endemic to hydrothermal vents and occupying different niches. These are the first sensory gene repertoires in Alvinocarididae, which enrich the relatively limited dataset available for crustaceans and, more broadly, for arthropods (Fig 5).

### Great diversity of IR

Transcriptomic analysis of the four alvinocaridid shrimp species revealed a remarkable diversity in chemosensory gene repertoires. An unexpected expansion of ionotropic receptors was observed in *Rimicaris exoculata*, with an estimated total of 442 IR sequences, and in *Mirocaris fortunata*, with a maximum total of 410 sequences. This diversity significantly exceeds that documented in other decapod crustaceans, including the 252 sequences in *Panulirus argus* [22] and the 337 IRs in the shrimp *Palaemon carinicauda* (present study). Gene duplication events have particularly occurred in the IR40a clade, with up to 94 sequences in *R. exoculata,* which significantly exceeds the number of 8 IR40a sequences found in the mantis shrimp *Oratosquilla oratoria* [80], and of 14 IR40a sequences found in *P. argus* [22]. Sensory systems exhibit plasticity and evolutionary adaptation in response to natural or anthropogenic environmental variations (see [81] and [82] for review). For example, the OR gene repertoires in vertebrates change depending on the living environment of each lineage, with a loss of a large number of ORs in primates with a well-developed visual system, and the expansion of ORs for airborne odors in the tetrapod lineage adapted to life on land [83]. With regard to aquatic lineages, marine tetrapods generally exhibit a reduction in the repertoire of chemoreceptors, and the repertoire of taste receptors correlates with the type of aquatic habitat in fish [84]. The observed IR expansion in *R. exoculata* and *M. fortunata* may result from ecological pressures driving the rapid diversification of chemosensory gene families. This pattern suggests that hydrothermal environments may require advanced sensory discrimination capabilities, due to a complex and variable chemical waterscape, where hydrothermal fluid mixes with the surrounding seawater, producing unstable and unique chemical signatures [85]. However, the IR repertoire showed notable differences between hydrothermal species, with diversity decreasing in the order *R. exoculata* > *M. fortunata* > *R. chacei* > *A. markensis*, which does not appear to correlate with the ecological niches of these species (Figs 1 and 3). These interspecific variations in the chemosensory gene repertoire suggest that *R. exoculata* and *M. fortunata* might have more developed chemosensory abilities. Given that these two species have distinct diets and microhabitats, the origin of this diversity remains unclear. *R. exoculata* is a primary consumer relying on symbiotic bacteria, living in dense swarms on high-temperature chimney walls with direct exposure to hot and turbulent vent fluids [9]. Conversely, *M. fortunata* is a predator/scavenger that inhabits cooler zones among mussel beds, where chemical signatures may be more spatially and temporally stable [16,55,56]. These ecological differences may have selected for differential expansion of IR clades, specialized for detection of distinct sets of chemicals.

Gene expansion related to deep-sea vent environments has already been described in the hydrothermal annelid *Alvinella pompejana*, which possesses an unusual diversity of respiratory enzymes and globin genes, possibly enabling it to better cope with the hypoxic conditions encountered at vent sites [86]. In deep-sea hydrothermal vent anemones, gene family expansions have also been reported, that are related to the stress response, including DNA repair and immune response, as well as to sulfide detoxification reactions [87]. The expansion of IRs in hydrothermal vent shrimps provides another striking example of gene family expansion in response to environmental selective pressures. These patterns align with broader evolutionary principles governing gene family evolution in extreme environments, where selection pressures favour the expansion of functionally critical gene families while allowing the contraction of less essential components [24,88].

Of the four conserved co-receptor IRs (IR25a, IR93a, IR8a and IR76b), three were expressed in the transcriptomes of antennules in all four hydrothermal shrimp, i.e., the protostome-conserved co-receptor IR25a, as well as the arthropod-conserved co-receptors IR8a and IR93a. Their expression patterns are consistent with those previously obtained for these species [12], as well as for other decapods [22]. In *R. exoculata*, the expression of these co-receptors was shown to be predominant in the lateral flagellum of the antennules, which is the primary chemosensory organ involved in olfaction in decapods [89]. Comparative studies on decapod crustaceans have consistently identified the lateral flagellum as the primary site of non-olfactory chemosensory neuron (CSN) concentration, and this flagellum also bear aesthetasc sensilla that contain multiple olfactory sensory neurons (OSNs) [19–21,90]. It is therefore expected that these co-receptors would be strongly expressed in this organ, which is rich in sensory neurons; however, further studies of their expression in different neuron types (CSNs or OSNs) will be necessary to determine their role in sensory detection. In *P. argus*, while all four co-receptors (IR25a, IR93a, IR8a and IR76b) were expressed in the lateral flagellum of the antennule, only the first two were found to be specifically expressed in OSNs [91]. It suggested that the co-receptors IR8a and IR76b are not involved in olfaction, and it remains to be determined in which types of neurons they are expressed and what sensory function they serve. No sequences of the co-receptor IR76b were found in the transcriptomes of the four hydrothermal species, which is unexpected considering that it is described as conserved among arthropods. This co-receptor exhibits a varied expression profile in decapod tissues, with different relative expression levels in the chemosensory organs (antennules, dactyls) of each species [22]. Unlike the other three co-receptors, it is therefore not thought to be specifically associated with olfaction but rather with chemoreception more broadly.

The localization of IR40a-family expression in the lateral flagellum of the antennule in *R. exoculata* may suggest a role for these IRs in olfaction. Indeed, members of the IR40a-family have been shown to be expressed in OSNs in *P. argus*, supporting the hypothesis of their involvement in olfaction [91]. The extensive diversification of this receptor family in hydrothermal shrimp may therefore result in a highly developed sense of smell in terms of the ability to discriminate between various odors. Further studies of their expression in neurons, their affinity for different ligands, and their function are nevertheless necessary to interpret this diversity.

### Thermal detection at vents

The environment of hydrothermal vents presents unique challenges in terms of thermal conditions, as they are the most thermally variable aquatic habitats, and the water can reach extreme temperatures exceeding the life limit of metazoans [76,92]. In [11], the authors have proposed that temperature serves as the primary navigational cue at vents, as both *R. exoculata* and *M. fortunata* proved to be attracted to warm water sources. Thus, thermal detection mechanisms are essential but remain to be described. Here, we identified TRP channels in the four hydrothermal shrimp species, which serve as multimodal sensory receptors and comprise families of thermosensitive channels [25,93]. All four species exhibited notably high diversity in the TRPA family (15–18 genes; Fig 4), which encompasses receptors sensitive to temperature, reactive oxygen species (H_2_O_2_), hydrogen sulfide and low pH commonly encountered in hydrothermal environments [93,94]. In comparison, only eight genes belonging to the TRPA family were identified in *P. argus* [22]. Since thermosensitive TRP channels exhibit distinct temperature activation ranges [95], this TRPA repertoire could be used to cover the temperature range in their habitats where the hydrothermal ﬂuid mixes with the surrounding seawater, which can vary abruptly from 2°C to over 30°C [96]. The expanded TRP channel repertoire in hydrothermal shrimp could also have critical roles in sensing and responding to oxidative and chemical hazards at vent environments. TRP channels have indeed been shown to play a role in chemical avoidance in another hydrothermal species, the annelid *Paralvinella hessleri*, and they have been suggested to be part of a hypersensitive detection system for acid and oxidative stress in this species [94]. The diversity of TRPA genes in shrimp may thus enable them to respond appropriately to different levels of oxidative stress, allowing them to avoid the most toxic areas and settle in optimal microhabitats.

The expression of some channels across all appendages, such as Pkd2 or Pain1, and the appendage-specific expression of other channels, such as Pain3 and TRPA1-like3, suggest functional specialization within the TRP channel repertoire of these appendages. The specific expression of TRPA1-like3 in the lateral flagellum, which is the primary olfactory tissue, potentially indicates integrated chemo- and thermosensory processing in this appendage. This expression pattern is consistent with that observed in *P. argus* and *Callinectes sapidus*, where TRPA1 is expressed at higher levels in the lateral flagellum of the antennule than in the dactyl [22], and corroborates the hypothesis that the antennule mediates temperature detection in crustaceans [19].

### Diversity of soluble binding proteins and gustatory receptors

Soluble proteins found in the olfactory organs of terrestrial arthropods, which can transport hydrophobic airborne odorants from the environment towards chemosensory receptors, might not be expected in aquatic crustaceans [89]. Although odorant-binding proteins (OBPs) are specific to hexapods, homologues of insect chemosensory proteins (CSPs), have been described in crustaceans [45,97]. They were generally found only in small numbers (0–4 per species), which led to the initial assumption that their involvement in chemical communication was unlikely [45,97]. A larger number of CSP sequences have since been discovered in a terrestrial isopod (13 CSPs identified in *Hemilepistus reaumurii*; [98]), and one CSP was recently described in the sea louse *Caligus rogercresseyi*, which was proven to be involved in host recognition through chemosensory detection [99]. We found between 4 and 8 CSP transcripts per species in the four hydrothermal shrimp, of which 6 sequences were overexpressed in the appendages (antennules, antennae, maxilliped and walking legs). Since these appendages harbor chemosensory and olfactory sensilla, these expression profiles suggest that CSPs may be involved in the transport of chemical compounds in hydrothermal shrimp. The CSP repertoire is poorly documented in crustaceans, and that of hydrothermal shrimp is currently the most diverse among aquatic crustaceans. Functional validation through protein-ligand binding assays, would definitively establish their roles.

The Niemann-Pick type C2 (NPC2) protein family has been suggested as a potential candidate for fulfilling the odorant transport role, based on their structural similarity and expression in the chemosensory organs of insects, arachnids and onychophorans [24,42,47,100–102]. In crustaceans, one NPC2 sequence has been identified in the prawn *Macrobrachium rosenbergii*, which is involved in cholesterol modulation in the male genital tract, similar to the role of NPC2s in vertebrates [103]. Three NPC2 sequences have also been described in the isopod *Hemilepistus reaumurii,* without any associated role [98]. We obtained 2–7 NPC2 sequences in deep-sea shrimp, and their expression profiles in all tissues tested (not restricted to olfactory organs) also did not allow any conclusions to be drawn about their potential role in chemical detection.

While this study documents the expansion of the IR, TRP and, to a lesser extent, CSP gene families, it also reveals contraction in the gustatory receptor (GR) family. The limited diversity of GRs (0–2 transcripts in the four hydrothermal species) is similar to that reported by [22] in four other decapods that have 1–4 GRs. This contrasts sharply with the considerable expansions of GRs found in the amphipod *Hyalella azteca* (155 GRs), in the copepod *Eurytempora affinis* (67 GRs) or in the branchiopod *Daphnia pulex* (59 GRs) [104]. The roles of GRs remain largely unknown in crustaceans, and the reduced number of these receptors in several species of decapods would appear to rule out them having a major role in chemical sensing in this group.

## Conclusion

The remarkable chemosensory adaptations reported in hydrothermal vent shrimp represent a compelling example of how life adapts to one of the most extreme environments on Earth. The high diversity of ionotropic receptors (IRs) and transient receptor potential (TRPs) channels demonstrates the evolutionary plasticity of sensory systems under intense selection pressure. The combined diversity of IRs and TRPs could enable hydrothermal shrimps to construct a rich multidimensional representation of the vent environment, where temperature gradients and chemical signatures indicate specific microhabitats suited to their particular ecological niches.

Added to these receptor diversifications, the sensory system of hydrothermal shrimp also exhibits highly developed brain zones, the hemiellipsoid bodies, that integrate sensory information from chemical, thermal or mechanical sensors [13]. The sensory gene expansions, in combination with the development of higher-order integrative brain regions, may facilitate navigation in heterogeneous and dynamic vent environments. This suggests that evolutionary responses to extreme environments involve system-level reorganization rather than discrete molecular changes.

## Supporting information

S1 Fig Heatmap showing the expression levels of IR transcripts in *A. markensis*, *M. fortunata,* and *R. chacei*; measured as transcripts per million (TPM) in three tissues (antennules, A1; second antennae, A2; abdominal muscle, Abd M). The raw TPM values are listed in S2 Table.
(PDF)

S2 Fig Phylogenetic tree and tissue expression of GRs in four hydrothermal shrimp. (A) Maximum-likelihood phylogeny of pancrustacean Gustatory Receptors (GRs), based on amino acid sequences identified in the transcriptomes of the hydrothermal shrimp species (*A. markensis*, Amar; *R. chacei*, Rcha; *R. exoculata*, Rexo) and in the genomes of the decapods *Cherax quadricarinatus* (Cqua), *Macrobrachium nipponense* (Mnip), *Penaeus chinensis* (Pchi), the water flea *D. pulex* (Dpul, Vizueta et al., 2020), and the fly *D. melanogaster* (Dmel, Vizueta et al., 2020). Colours represent different groups of orthologs containing sequences from hydrothermal species. The scale bar shows the expected number of amino acid substitutions per site. (B) Heatmap showing expression levels of GR transcripts, measured as transcripts per million (TPM) in six tissues for *R. exoculata* (medial flagellum of the antennules, A1 MF; lateral flagellum of the antennules, A1 LF; second antennae, A2; mix of second and third maxillipeds plus first walking legs, Mxp2,3/P1; fifth walking legs, P5; abdominal muscle, Abd M) and in three tissues for *A. markensis* and *R. chacei* (antennules, A1; second antennae, A2; abdominal muscle, Abd M). Raw TPM values are available in S2 Table.
(PDF)

S3 Fig Phylogenetic tree and tissue expression of CSPs in four hydrothermal shrimp. (A) Maximum-likelihood phylogeny of pancrustacean Chemosensory Proteins (CSPs), based on amino acid sequences identified in the transcriptomes of the hydrothermal shrimp species (*A. markensis*, Amar; *M. fortunata*, Mfor; *R. chacei*, Rcha; *R. exoculata*, Rexo) and in the genomes of the decapods *C. quadricarinatus* (Cqua), *M. nipponense* (Mnip), *P. chinensis* (Pchi), the water flea *D. pulex* (Dpul, Vizueta et al., 2020) and the fly *D. melanogaster* (Dmel, Vizueta et al., 2020). Colours represent different groups of orthologs containing sequences from hydrothermal species. The scale bar shows the expected number of amino acid substitutions per site. (B) Heatmap showing the expression levels of CSP transcripts, measured as transcripts per million (TPM), in six tissues of *R. exoculata* and three tissues of the other species (as described in S2 Fig). Raw TPM values are available in S2 Table.
(PDF)

S4 Fig Phylogenetic tree and tissue expression of NPC2s in four hydrothermal shrimp. (A) Maximum-likelihood phylogeny of pancrustacean Niemann-Pick type C2 (NPC2) proteins based on amino acid sequences identified in the transcriptomes of the hydrothermal shrimp species (*A. markensis*, Amar; *M. fortunata*, Mfor; *R. chacei*, Rcha; *R. exoculata*, Rexo) and in the genomes of the decapods *C. quadricarinatus* (Cqua), *M. nipponense* (Mnip), *P. chinensis* (Pchi), the water flea *D. pulex* (Dpul, Vizueta et al., 2020), and the fly *D. melanogaster* (Dmel, Vizueta et al., 2020). Colours represent different groups of orthologs containing sequences from hydrothermal species. The scale bar shows the expected number of amino acid substitutions per site. (B) Heatmap showing the expression levels of NPC2 transcripts, measured as transcripts per million (TPM), in six tissues of *R. exoculata* and three tissues of the other species (as described in S2 Fig). Raw TPM values are available in S2 Table.
(PDF)

S5 Fig Tissue expression of TRPs in three hydrothermal shrimp. Heatmap showing expression levels of TRP transcripts in *A. markensis*, *M. fortunata,* and *R. chacei*, measured as transcripts per million (TPM) in three tissues (as described in S1 Fig). Raw TPM values are available in S2 Table.
(PDF)

S1 Table Summary of raw data and transcriptome assembly metrics for the four shrimp species used in this study. A1 MF: medial flagellum of the antennule; A1 LF: lateral flagellum of the antennule; A2: second antenna; Mxp2,3: second and third pairs of maxillipeds; P1: first pair of walking legs; P5: fifth pair of walking legs. Abd M: abdominal muscle.
(PDF)

S2 Table Raw values for sensory gene expression, measured as transcripts per million (TPM), for the four shrimp species used in this study. (XLSX)
