# Supplementary figures and images for: The sensory gene repertoire of deep-sea hydrothermal shrimp
Source: PLoS One. 2026 Jul 15;21(7):e0354016. doi: 10.1371/journal.pone.0354016 (PMC13372173; doi:10.1371/journal.pone.0354016)

*Rimicaris chacei*

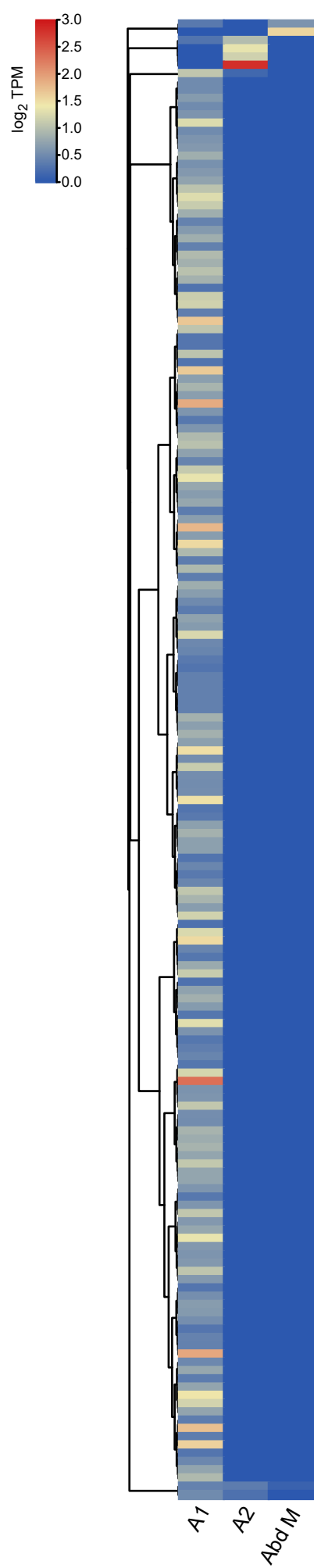

*Mirocaris fortunata*

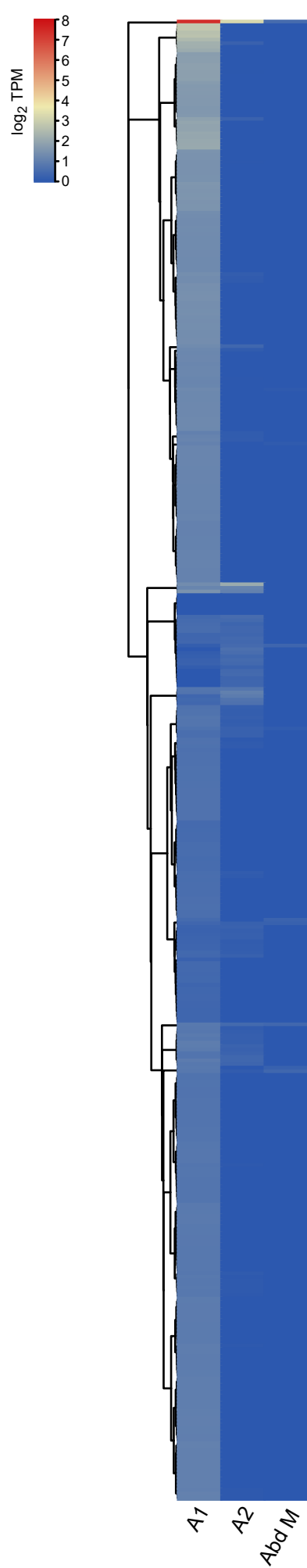

*Alvinocaris markensis*

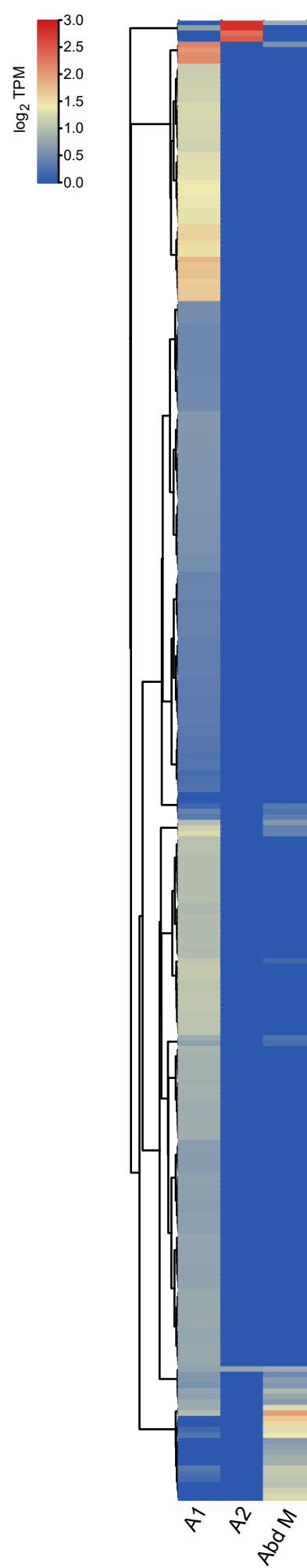

Supplement: S1 Fig — The raw TPM values are listed in S2 Table. (PDF) [file pone.0354016.s001.pdf]

**A**

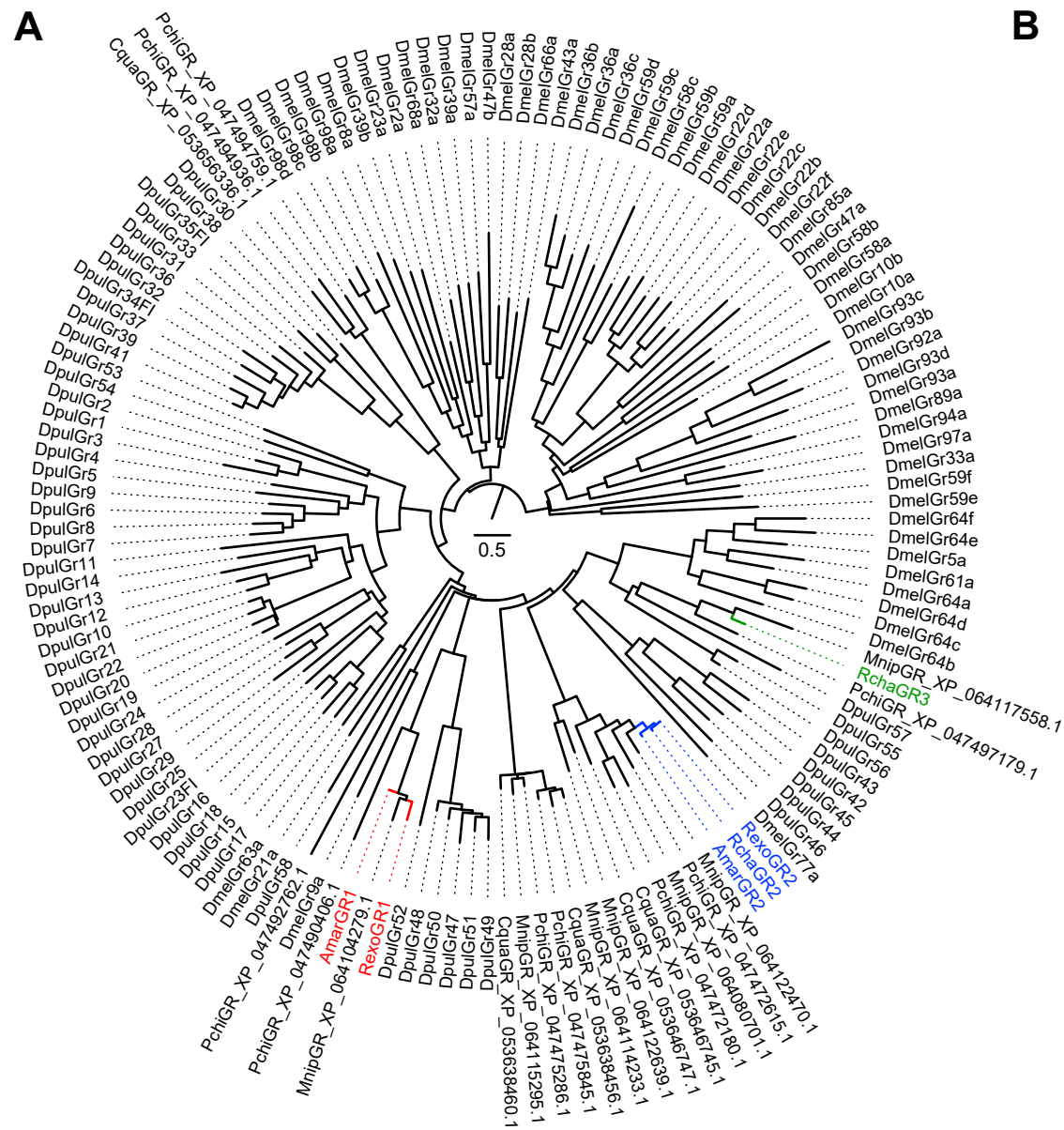

**B**

*Rimicaris exoculata*

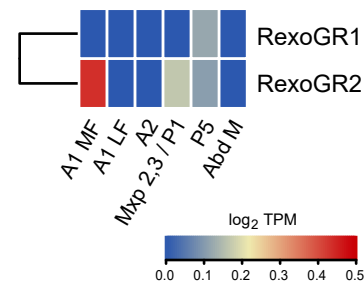

*Rimicaris chacei*

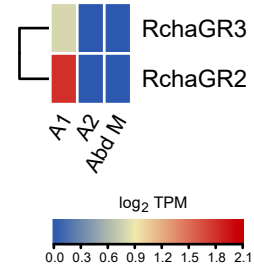

*Alvinocaris markensis*

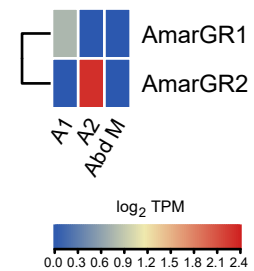

Supplement: S2 Fig — (A) Maximum-likelihood phylogeny of pancrustacean Gustatory Receptors (GRs), based on amino acid sequences identified in the transcriptomes of the hydrothermal shrimp species (A. markensis, Amar; R. chacei, Rcha; R. exoculata, Rexo) and in the genomes of the decapods Cherax quadricarinatus (Cqua), Macrobrachium nipponense (Mnip), Penaeus chinensis (Pchi), the water flea D. pulex (Dpul, Vizueta et al., 2020), and the fly D. melanogaster (Dmel, Vizueta et al., 2020). Colours represent different groups of orthologs containing sequences from hydrothermal species. The scale bar shows the expected number of amino acid substitutions per site. (B) Heatmap showing expression levels of GR transcripts, measured as transcripts per million (TPM) in six tissues for R. exoculata (medial flagellum of the antennules, A1 MF; lateral flagellum of the antennules, A1 LF; second antennae, A2; mix of second and third maxillipeds plus first walking legs, Mxp2,3/P1; fifth walking legs, P5; abdominal muscle, Abd M) and in three tissues for A. markensis and R. chacei (antennules, A1; second antennae, A2; abdominal muscle, Abd M). Raw TPM values are available in S2 Table. (PDF) [file pone.0354016.s002.pdf]

**A**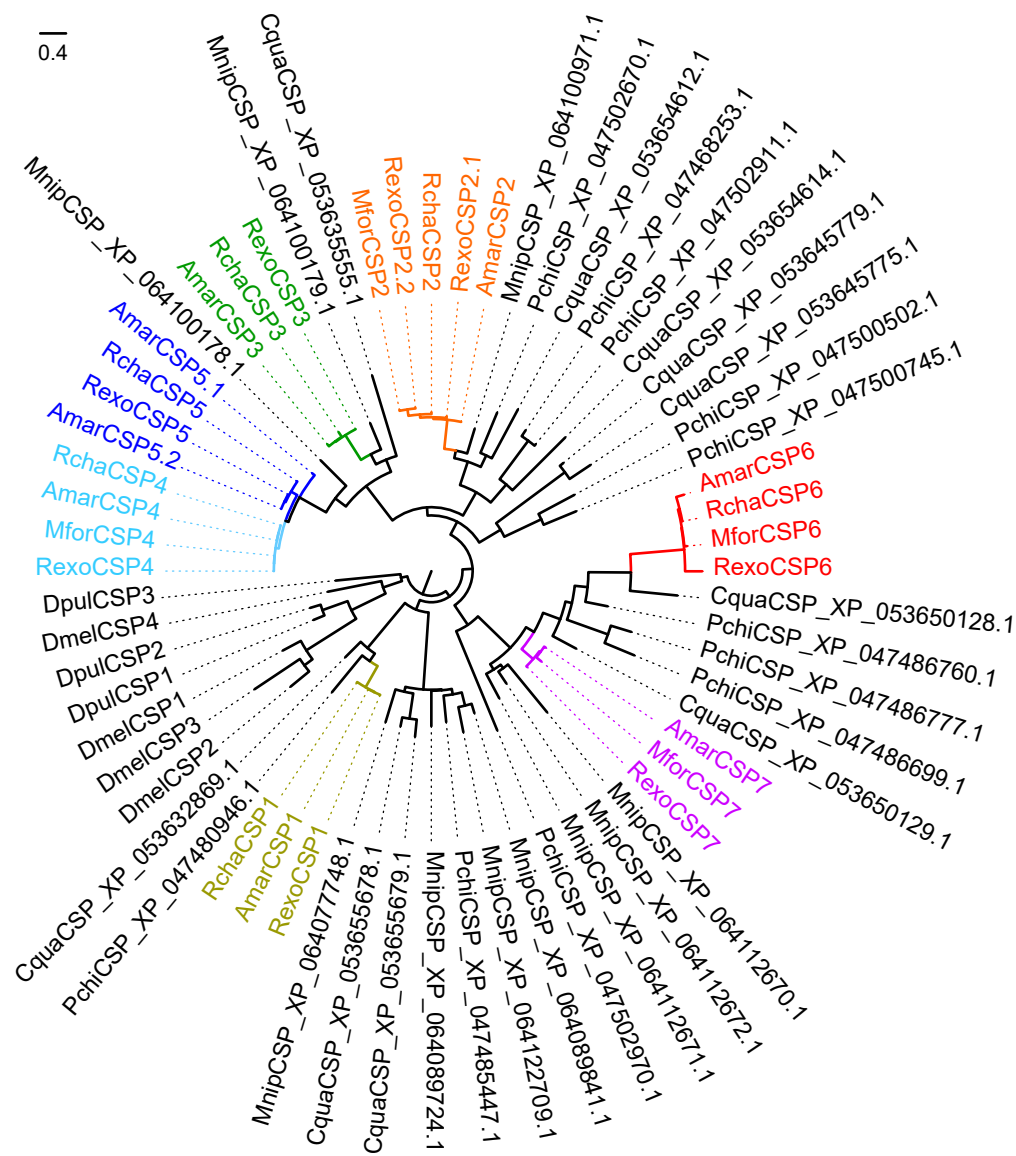**B***Rimicaris exoculata*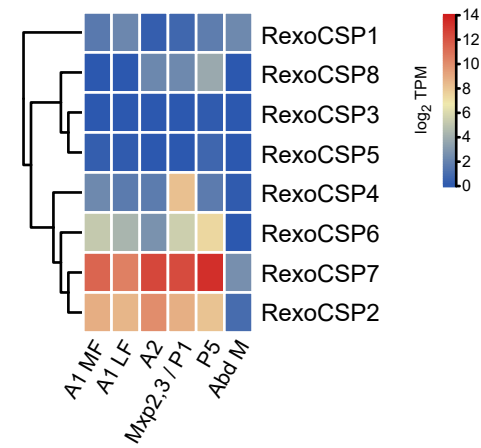*Rimicaris chacei*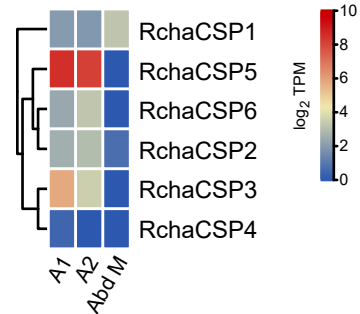*Alvinocaris markensis*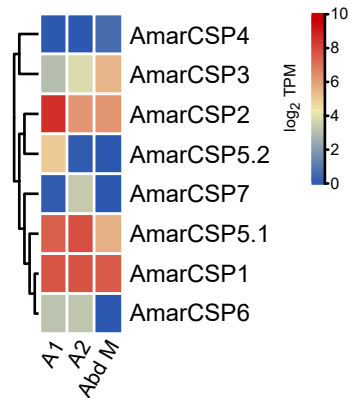*Mirocaris fortunata*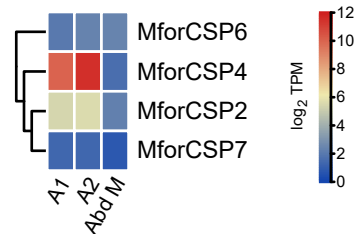

Supplement: S3 Fig — (A) Maximum-likelihood phylogeny of pancrustacean Chemosensory Proteins (CSPs), based on amino acid sequences identified in the transcriptomes of the hydrothermal shrimp species (A. markensis, Amar; M. fortunata, Mfor; R. chacei, Rcha; R. exoculata, Rexo) and in the genomes of the decapods C. quadricarinatus (Cqua), M. nipponense (Mnip), P. chinensis (Pchi), the water flea D. pulex (Dpul, Vizueta et al., 2020) and the fly D. melanogaster (Dmel, Vizueta et al., 2020). Colours represent different groups of orthologs containing sequences from hydrothermal species. The scale bar shows the expected number of amino acid substitutions per site. (B) Heatmap showing the expression levels of CSP transcripts, measured as transcripts per million (TPM), in six tissues of R. exoculata and three tissues of the other species (as described in S2 Fig). Raw TPM values are available in S2 Table. (PDF) [file pone.0354016.s003.pdf]

**A**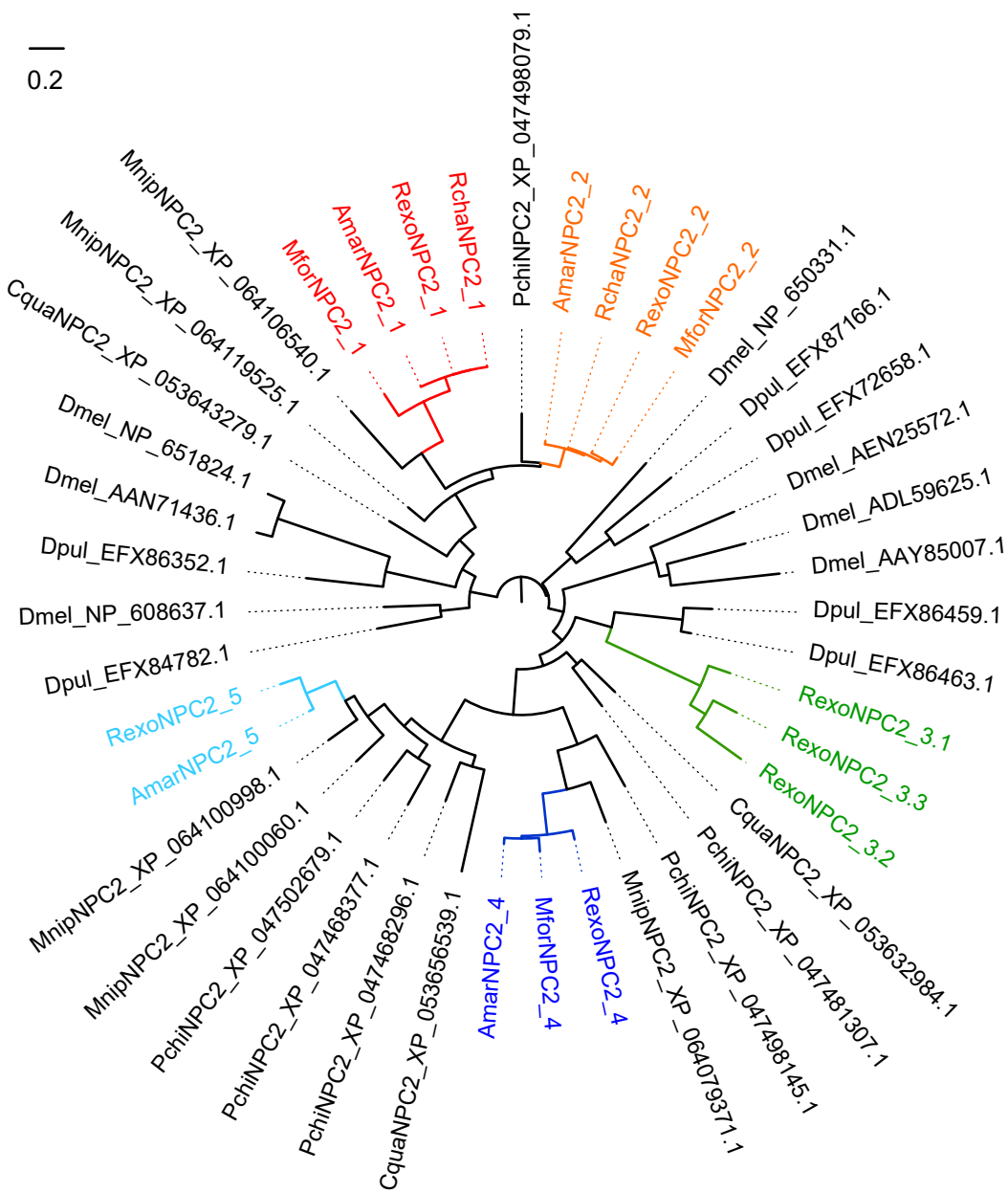**B**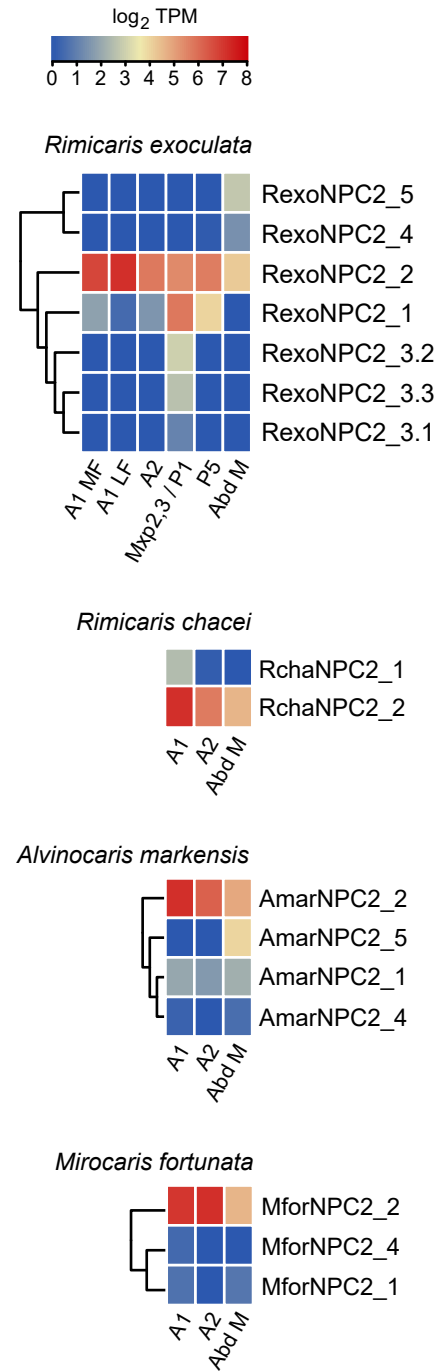

Supplement: S4 Fig — (A) Maximum-likelihood phylogeny of pancrustacean Niemann-Pick type C2 (NPC2) proteins based on amino acid sequences identified in the transcriptomes of the hydrothermal shrimp species (A. markensis, Amar; M. fortunata, Mfor; R. chacei, Rcha; R. exoculata, Rexo) and in the genomes of the decapods C. quadricarinatus (Cqua), M. nipponense (Mnip), P. chinensis (Pchi), the water flea D. pulex (Dpul, Vizueta et al., 2020), and the fly D. melanogaster (Dmel, Vizueta et al., 2020). Colours represent different groups of orthologs containing sequences from hydrothermal species. The scale bar shows the expected number of amino acid substitutions per site. (B) Heatmap showing the expression levels of NPC2 transcripts, measured as transcripts per million (TPM), in six tissues of R. exoculata and three tissues of the other species (as described in S2 Fig). Raw TPM values are available in S2 Table. (PDF) [file pone.0354016.s004.pdf]

*Rimicaris chacei*

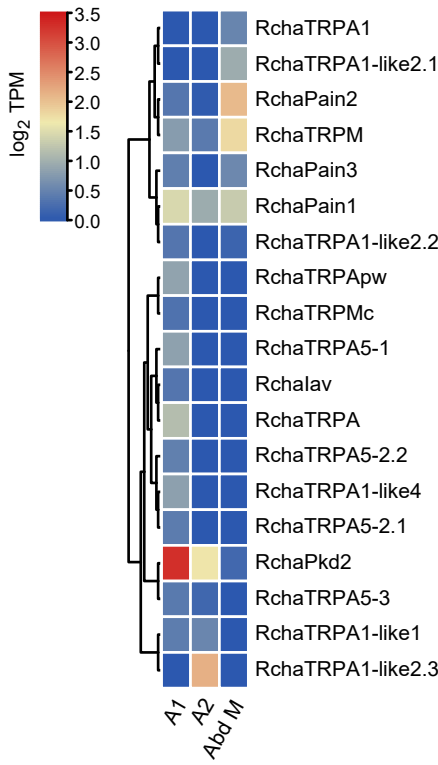

*Mirocaris fortunata*

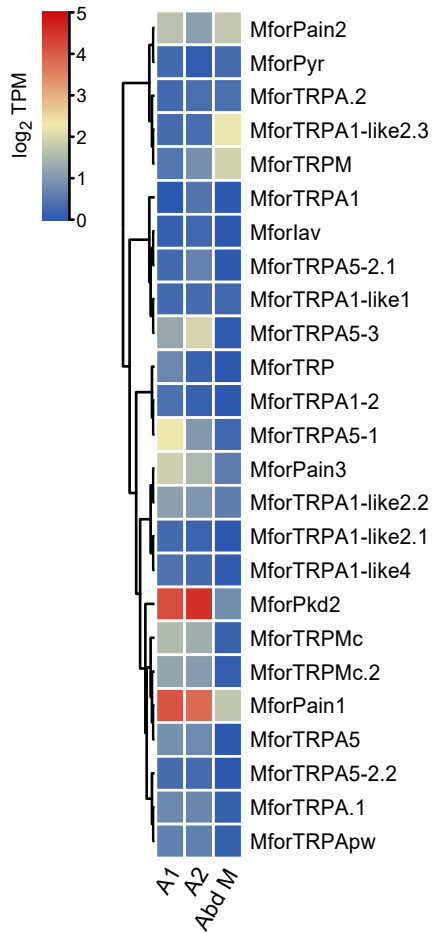

*Alvinocaris markensis*

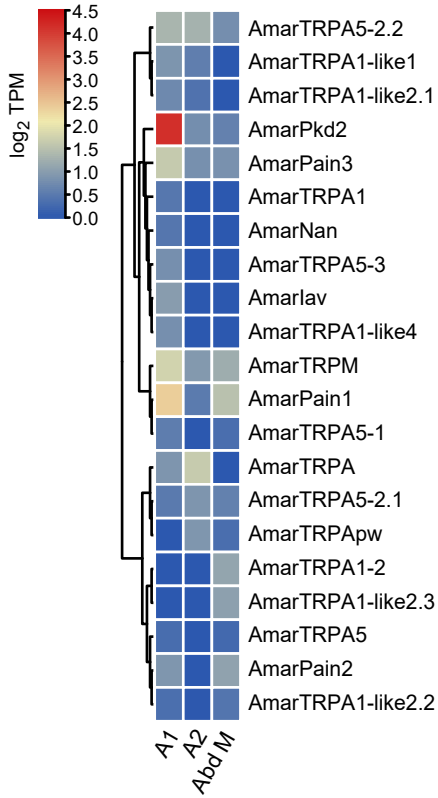

Supplement: S5 Fig — Heatmap showing expression levels of TRP transcripts in A. markensis, M. fortunata, and R. chacei, measured as transcripts per million (TPM) in three tissues (as described in S1 Fig). Raw TPM values are available in S2 Table. (PDF) [file pone.0354016.s005.pdf]
