# Supplementary material for: The sensory gene repertoire of deep-sea hydrothermal shrimp
Source: PLoS One. 2026 Jul 15;21(7):e0354016. doi: 10.1371/journal.pone.0354016 (PMC13372173; doi:10.1371/journal.pone.0354016)
Supplement: S1 Table — A1 MF: medial flagellum of the antennule; A1 LF: lateral flagellum of the antennule; A2: second antenna; Mxp2,3: second and third pairs of maxillipeds; P1: first pair of walking legs; P5: fifth pair of walking legs. Abd M: abdominal muscle. (PDF) [file pone.0354016.s006.pdf]

S1 Table. Summary of raw data and transcriptome assembly metrics for the four shrimp species used in this study. A1 MF: medial flagellum of the antennule; A1 LF: lateral flagellum of the antennule; A2: second antenna; Mxp2,3: second and third pairs of maxillipeds; P1: first pair of walking legs; P5: fifth pair of walking legs. Abd M: abdominal muscle.

Species	Sample	Total number of raw reads	Total number of cleaned reads	Numb. of contigs	Number of unigenes	Complete BUSCO genes (%)	Single-copy BUSCO (%)
Rimicaris exoculata	A1 MF	20,366,936	20,226,159	514,082	104,607	89.7	82.5
	A1 LF	24,659,932	23,586,189				
	A2	19,998,203	19,056,886				
	Abd M	68,514,035	67,313,780				
	Mxp2,3 and P1	20,364,994	19,918,823				
	P5	80,297,772	79,070,490				
Rimicaris chacei	A1 MF and LF	16,102,153	15,276,667	224,046	71,225	72.5	70.1
	A2	25,430,056	21,105,346				
	Abd M	17,076,640	16,765,972				
Alvinocaris markensis	A1 MF and LF	18,062,170	17,532,808	303,257	90,309	77.1	74.4
	A2	28,594,677	27,081,706				
	Abd M	7,370,076	7,306,016				
Mirocaris fortunata	A1 MF and LF	10,320,910	10,252,121	621,742	128,893	82.2	79.2
	A2	34,645,408	34,362,241				
	Abd M	17,514,469	17,361,892				
